# Physiological and biochemical responses of strawberry crown and leaf tissues to freezing stress

**DOI:** 10.1186/s12870-021-03300-2

**Published:** 2021-11-13

**Authors:** Elnaz Zareei, Farhad Karami, Mansour Gholami, Ahmad Ershadi, Saber Avestan, Rishi Aryal, Gholamreza Gohari, Muhammad Farooq

**Affiliations:** 1 Department of Horticultural Science, Faculty of Agriculture, University of Tabriz, Tabriz, Iran; 2 Horticultural Research Department, Kurdistan Agricultural and Natural Resources Research and Education Center, AREEO, Sanandaj, Iran; 3 Department of Horticultural Sciences, Faculty of Agriculture, Bu-Ali Sina University, Hamedan, Iran; 4 Department of Horticultural Science, NC State University, Raleigh, NC USA; 5 Department of Horticultural Sciences, Faculty of Agriculture, University of Maragheh, Maragheh, Iran; 6 Department of Plant Sciences, College of Agricultural and Marine Sciences, Sultan Qaboos University, 123 Al-Khoud, Oman

**Keywords:** Freezing injury, TBARS, Strawberry, Fv/Fm, Cold acclimation, SOD, Abiotic stress

## Abstract

**Background:**

In northern Iran and other cold regions, winter freezing injury and resultant yield instability are major limitations to strawberry production. However, there is scarcity of information on the physiological and biochemical responses of strawberry cultivars to freezing stress. This study aimed to investigate the physiological and biochemical responses of strawberry cultivars (Tennessee Beauty, Blakemore, Kurdistan, Queen Elisa, Chandler, Krasnyy Bereg, and Yalova) to different freezing temperature treatments (− 5, − 10, − 15, − 20, and − 25 °C) under controlled conditions.

**Results:**

All measured physiological and biochemical features were significantly affected by the interaction effect between low temperatures and cultivars. Tennessee Beauty showed the highest RWC at − 25 °C. The highest Fv/Fm was observed in Queen Elisa. Krasnyy Bereg had the least freezing injury (FI) in crown and leaf, while Yalova and Chandler showed the highest crown and leaf FI, respectively. At − 20 to − 25 °C, the highest carbohydrates contents of crown and leaf were noted in Blakemore and Krasnyy Bereg cultivars, respectively. The Yalova showed the highest protein content in both crown and leaf tissues at − 25 °C. The Tennessee Beauty and Blackmore cultivars showed the highest proline in crowns and leaves at − 15 °C, respectively. The highest ThioBarbituric Acid Reactive Substances (TBARS) contents in the crown and leaf were observed in Kurdistan and Queen Elisa, respectively. Queen Elisa and Krasnyy Bereg cultivars showed SOD and POD peaks in the crown at − 15 °C, respectively.

**Conclusion:**

Freezing stress was characterized by decreased Fv/Fm and RWC, and increased FI, TBARS, total carbohydrates, total proteins, proline content, and antioxidant enzyme activity. The extent of changes in above mentioned traits was cultivar dependent. FI and TBARS were the best traits among destructive parameters for evaluating freezing tolerance. Moreover, maximum quantum yield of PSII (Fv/Fm index), as non-destructive parameters, showed a significant efficiency in rapid assessment for screening of freezing tolerant strawberry cultivars. The cultivars Krasnyy Bereg, Queen Elisa, and Kurdistan were the most tolerant cultivars to freezing stress. These cultivars can be used as parents in breeding programs to develop new freezing tolerant cultivars.

**Supplementary Information:**

The online version contains supplementary material available at 10.1186/s12870-021-03300-2.

## Background

Freeze injury is one of the major environmental factors limiting the growth, development, and productivity of plants in temperate regions. The freeze injury depends on mechanisms linking cell dehydration and membrane disintegration via water crystallization during freezing [1–3]. However, the plant responses to freezing stress and associated tolerance mechanism involves different physiological, molecular, and metabolic alterations and adaptations [4, 5]. These include the accumulation of soluble sugars, amino acids, cold-induced stress-related proteins..This expression of encoded genes to stabilize membranes against injury is an important component of this freezing tolerance mechanism [6, 7].

The plant’s ability to tolerate low temperatures under natural conditions vary with plant species, cultivars, and tissues [2, 8, 9]. However, evaluation of freezing injury is significantly crucial for predicting plant winter survival, regrowth in the following spring. Such evaluation is also desired to recognize freezing-tolerant species and cultivars and to develop management strategies to improve plant performance under freezing stress. Strawberry (*Fragaria × ananassa* Duchesne) is a widely grown hybrid species of the genus Fragaria, collectively known as the strawberries. Strawberries are grown widely from mild maritime to severe temperate continental climates throughout the world. Despite its wider adaptability, the freezing stress is a major limiting factor in the production of strawberries particularly in the temperate regions. The changing climate is expected to increase in the extreme temperature events and associated spatio-temporal patterns [10]. These temperature extremes have a significant effect on the growth and productivity of strawberries [11, 12].

This is interesting to note that plant exposure to non-freezing low temperatures can improve tolerance to freezing temperate plants through cold acclimation [13, 14]. Strawberry plants also acclimatize to freezing temperature conditions and can survive freezing temperatures by tolerating ice crystal formation in crown tissues. Water movement from within the cell to the intercellular spaces where ice crystals are formed [15, 16]. This mechanism in association with osmotic adjustment help plants improve cold tolerance. In this regard, selection and use of cultivars with potential to tolerate environmental changes are crucial for surviving at freezing temperatures. Conferring to the qualified advantages and economic returns of strawberry production, it is necessary to identify appropriate cold tolerant cultivars as an advanced content of a given metabolite might associate with higher probabilities of survival before developmental programs. Although several studies report the mechanism of freezing stress tolerance in different plant species, studies on the physiological and biochemical responses of strawberry plants to low temperature stress and associated tolerance mechanisms are lacking. Moreover, the information on genotypic variability among different strawberry cultivars in response to low temperature stress in also lacking. This study was, therefore, conducted to evaluate the physiological and biochemical indicators reflecting the plant responses to freezing stress in different strawberry cultivars and identify the freezing tolerant cultivars. This was hypothesized that strawberry cultivars differ in their responses to low temperature stress.

## Results

The analysis of variance indicates that freezing temperature treatments, cultivars, and the interaction of freezing temperatures and cultivars significantly affected all physiological and biochemical traits (Table 1).

**Table 1:** Analysis of variance for the influence of freezing temperature stress on physiological, biochemical, and morphological traits of strawberry cultivars

		**Mean sum of squares**							
**Source of variance**	df	F_v_/F_m_	RWC	Leaf FI	Crown FI	Leaf total soluble carbohydrates	Crown total soluble carbohydrates	Crown total protein	Leaf total protein
**Temperature (T)**	5	0.24**	2672.32**	7033.60**	13,085.56**	1559.10**	1169.19**	605.43**	1017.92**
**Cultivar (C)**	6	0.041**	156.07**	841.38**	281.68**	96.99**	124.54**	44.83**	101.13**
**T × C**	30	0.006**	62.93**	180.20**	183.5**	13.38**	20.47**	35.56**	21.99**
**Error**	84	0.0001	10.66	3.07	0.97	2.79	0.34	1.43	0.64
**Coefficient of variation (%)**		2.79	4.78	9.73	4.69	3.94	3.27	7.81	4.27
		**Mean sum of squares**							
**Source of variance**	df	Leaf proline	Crown proline	Leaf TBARS	Crown TBARS	Leaf SOD	Crown SOD	Leaf POD	Crown POD
**Temperature (T)**	5	18.39**	21.99**	515.82**	513.90**	1637.38**	238.45**	98.37**	1076.53**
**Cultivar (C)**	6	2.57**	2.57**	28.75**	40.25**	240.07**	33.86**	73.49**	219.16**
**T × C**	30	1.85**	1.16**	7.53**	8.86**	134.98**	81.88**	76.19**	166.13**
**Error**	84	0.02	0.009	0.61	0.56	1.17	0.87	2.49	3.07
**Coefficient of variation (%)**		9.35	5.93	6.35	6.41	8.77	10.19	13.23	16.67

*df* Degree of freedom, *Fv/Fm* Maximum quantum yield of PSII, *RWC* Leaf relative water content, *FI* Freezing injury, *TBARS* Thiobarbituric acid reactive substances, *SOD* Superoxide dismutase, *POD* Peroxidase

** Significant at *P* ≤ 0.05

### Maximum quantum yield of PSII (Fv/Fm)

**Fig. 1:**
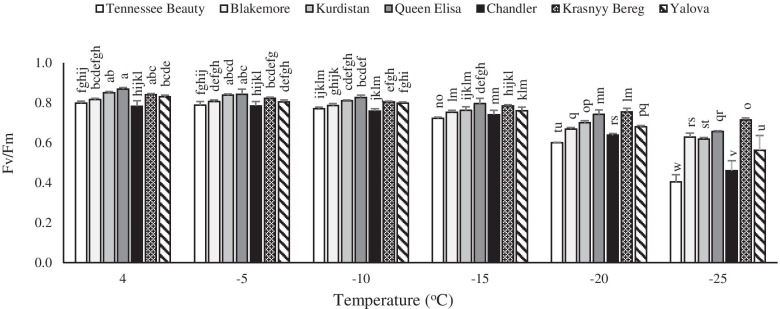
Effects of freezing temperature treatments on maximum quantum yield of PSII (Fv/Fm) in leaf tissues of different strawberry cultivars. Data points are means of three replicates and vertical bars indicate standard deviations at *P ≤ 0.05*

A decrease in the leaf Fv/Fm was observed with a decrease in temperature in all tested strawberry cultivars. The highest reduction in Fv/Fm was observed in the cultivars Tennessee Beauty and Chandler under freezing stress, especially at − 15 °C to − 25 °C (Fig. 1). The Fv/Fm was decreased up to 50 and 41% in cultivars Tennessee Beauty and Chandler compared to the controls, respectively (Fig. 1).

### Leaf relative water contents (RWC)

**Fig. 2:**
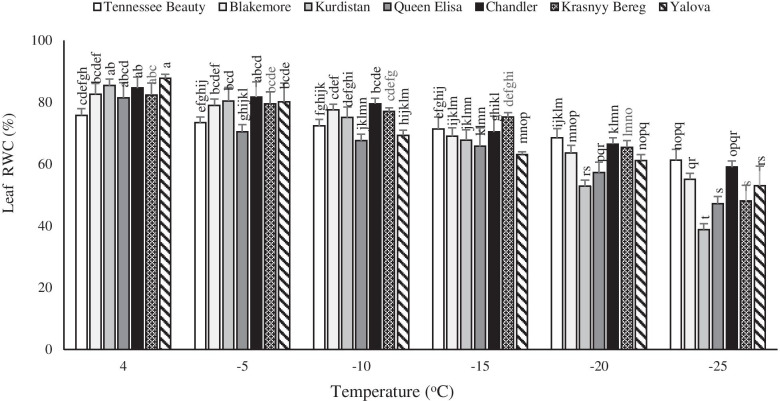
Effects of freezing temperature treatments on leaf relative water content (RWC) in leaf tissue of different strawberry cultivars. Data points are means of three replicates and vertical bars indicate standard deviations at *P ≤ 0.05*

The leaf relative water contents decreased significantly by low temperature treatment in all tested strawberry cultivars. However, the rate of decrease differed among the tested cultivars. The cultivar Tennessee Beauty had the RWC value of 75.8% at + 4 °C, the lowest value at this temperature. At − 25 °C, the cultivar Tennessee Beauty had the highest RWC of 61.24% compared to the lowest value of 38.5% in the cultivar Kurdistan. The highest RWC was noted in the cultivar Yalova at + 4 °C compared to the other cultivars, while the lowest value was observed in the cultivar Kurdistan at − 25 °C (Fig. 2). The rate of reduction of RWC content in the cultivars Queen Elisa, Krasnyy Bereg, and Kurdistan under freezing temperatures was higher than that of the cultivars Chandler, Tennessee Beauty, and Blackmore (Fig. 2).

### Freezing injury (FI)

**Fig. 3:**
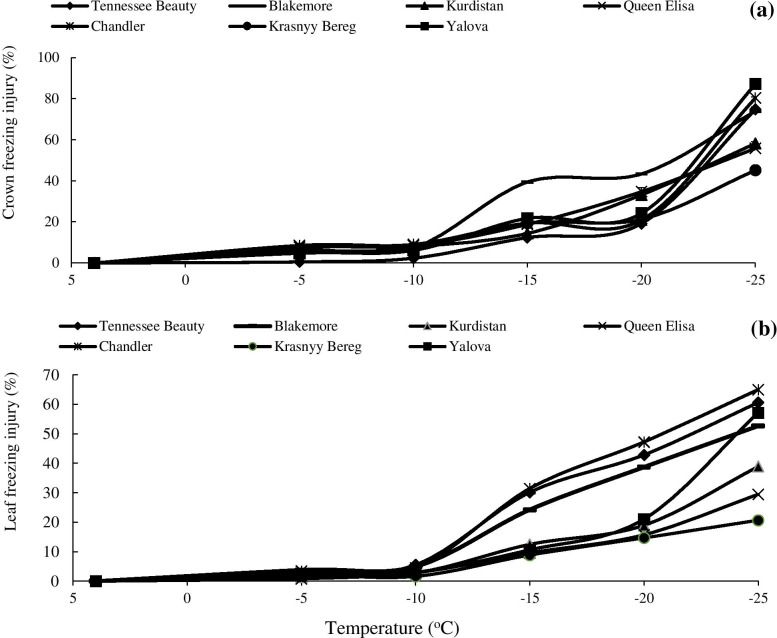
Effects of freezing temperature treatments on freezing injury in the crown (**a**) and leaf (**b**) tissues of different strawberry cultivars. Data points are means of replicates (*n* = 3) and vertical bars indicate standard deviations at *P ≤ 0.05*

The rate of freezing injury (FI) in the crown and leaf tissues increased with a decrease in temperature in all tested strawberry cultivars (Fig. 3; Fig. S1). At − 15 °C, a significant increase in crown FI was observed in the cultivar Blakmore compared to the other cultivars (Fig. 3a). However, the highest FI was observed in the cultivar Yalova followed by the cultivars Chandler and Tennessee Beauty with decreasing the temperature to − 25 °C. The range of differences between cultivars in terms of crown FI was higher at − 25 °C than other freezing temperatures. Therefore, the cultivars were divided into two groups: the first group had cultivars Krasnyy Bereg, Queen Elisa, and Kurdistan with a low rate of FI, and the cultivars Yalova, Chandler, Tennessee Beauty, and Blackmore with high crown FI were placed in the second group (Fig. 3a). Leaf FI in all cultivars was zero at + 4 °C, while an increase in FI was noticed with decrease in the temperature (Fig. 3b). With a decrease in the temperature to − 15 °C, a significant increase in FI was noted in the cultivars Chandler, Tennessee Beauty, and Blackmore compared to other cultivars. The cultivars Krasnyy Bereg, Queen Elisa, and Kurdistan had the lowest leaf FI at − 15 °C (between 8 to 12%). However, the relative FI remained between 53 to 65% in the other four cultivars (Fig. 3b).

### Total soluble carbohydrates

**Fig. 4:**
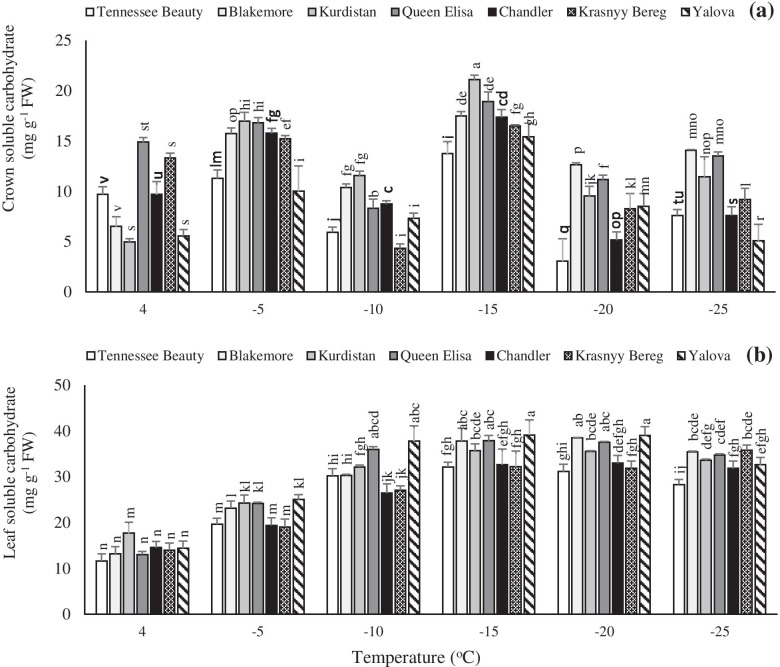
Effects of freezing temperature treatments on soluble carbohydrate in the crown (**a**) and leaf (**b**) tissues of different strawberry cultivars. Data points are means of three replicates and vertical bars indicate standard deviations at *P ≤ 0.05*

Before the imposition of freezing temperature treatments (at + 4 °C), crowns of cultivars Queen Elisa and Krasnyy Bereg showed the highest amount of total soluble carbohydrates compared to other cultivars. The highest crown soluble carbohydrates contents were recorded in the cultivar Kurdistan at − 5 to − 15 °C, while the cultivar Blackmore had the highest total soluble carbohydrates at temperatures − 20 to − 25 °C. The cultivar Tennessee Beauty had the lowest carbohydrates contents at the most temperature ranges (Fig. 4a). The cultivar Kurdistan had the highest leaf carbohydrates contents compared to other cultivars at + 4 °C. Between − 5 to − 20 °C, the highest leaf carbohydrate contents were noted in the cultivar Yalova. At − 25 °C, the cultivars Krasnyy Bereg and Tennessee Beauty had the highest and lowest leaf carbohydrates contents, respectively (Fig. 4b). The response of strawberry plants to freezing temperature showed a significant difference in terms of leaf carbohydrates. In contrast to other cultivars, the cultivar Krasnyy Bereg maintained an upward trend up to − 25 °C. As temperature decreased from − 20 to − 25 °C, a rapid and significant increase in the soluble carbohydrates was noted in this cultivar. However, in other cultivars, the maximum level of soluble carbohydrates was detected in the temperature range from − 15 to − 20 °C. However, a reduction in these values was noted with a decrease in temperature (Fig. 4b).

### Total soluble protein

**Fig. 5:**
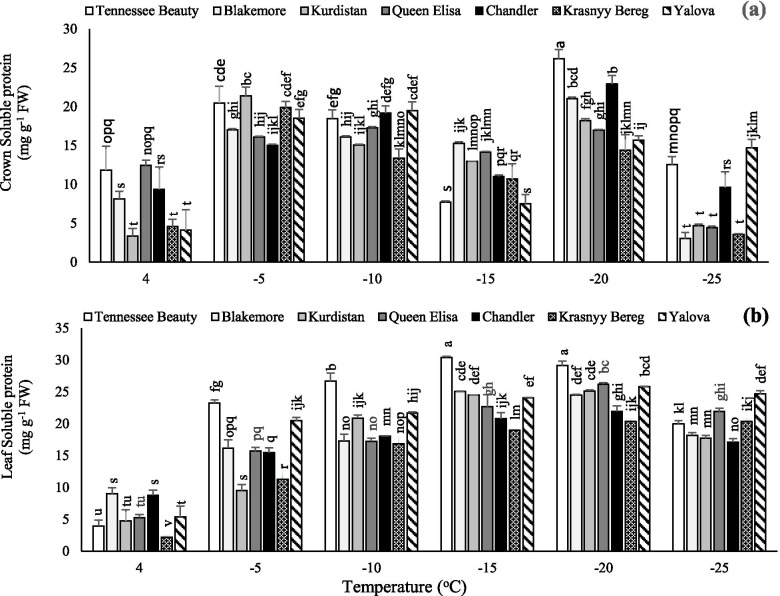
Effects of freezing temperature treatments on soluble protein in the crown (**a**) and leaf (**b**) tissues of different strawberry cultivars. Data points are means of three replicates and vertical bars indicate standard deviations at *P ≤ 0.05*

A decrease in temperature to − 5 °C caused a significant increase in the total soluble protein of crown tissue in all tested cultivars (Fig. 5). Then, up to − 15 °C, a decreasing trend was observed in the soluble protein. At − 20 °C, a rapid increment in the crown soluble protein was noted in all tested strawberry cultivars. However, this increase at − 20 °C temperature was much higher in sensitive cultivars including Tennessee Beauty and Chandler (Fig. 5a). Although, the total protein peak in crowns was observed in the sensitive and tolerant cultivars at − 20 and − 5 °C, respectively (Fig. 5a), sensitive cultivars including Tennessee Beauty and Chandler had the highest total soluble protein contents in the crown tissue compared to other cultivars at − 20 °C, respectively. While the lowest total soluble protein contents were noted in the tolerant cultivar Krasnyy Bereg at − 25 °C. The highest leaf soluble protein contents were observed in the cultivar Tennessee Beauty at − 15 °C (Fig. 5b). In general, the sensitive cultivar Tennessee Beauty had the highest leaf total protein in comparison with other cultivars at the temperature range of − 5 to − 20 °C. However, the least changes were observed in Krasnyy Bereg in contrast to the other tested cultivars. In this cultivar, no decreasing trend of soluble proteins was noted at the temperature range of − 20 to − 25 °C (Fig. 5).

### Proline content

**Fig. 6:**
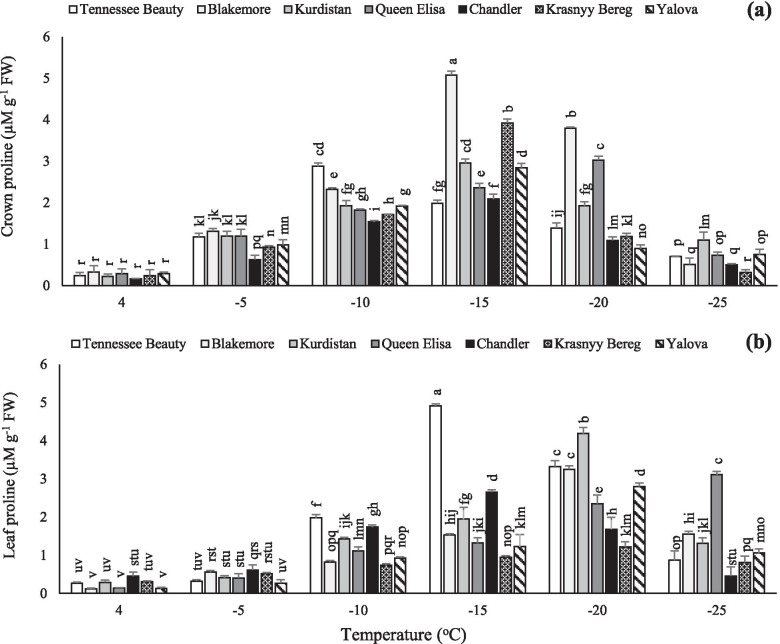
Effects of freezing temperature treatments on proline in the crown (**a**) and leaf (**b**) tissues of different strawberry cultivars. Data points are means of three replicates and vertical bars indicate standard deviations at *P ≤ 0.05*

A significant increase in the crown proline was noted in the cultivar Tennessee Beauty than other cultivars with the temperature decrease to − 10 °C. The highest crown proline was noted at − 15 °C and − 20 °C for the cultivar Blakemore (Fig. 6). A decreasing trend in crown proline content was observed in all tested cultivars at − 25 °C (Fig. 6a). With a decrease in temperature, an increase in leaf proline content was noted in all tested strawberry cultivars. Although, the highest proline contents in the tested cultivars were noted at different temperatures (Fig. 6b), the highest leaf proline contents in cultivars Tennessee Beauty and Chandler were noted at − 15 °C (earlier than other tested cultivars) due to more sensitivity to the low temperatures. The highest leaf proline contents were observed in the cultivars Blakemore, Kurdistan, Krasnyy Bereg, and Yalova at − 20 °C, and Queen Elisa at − 25 °C (Fig. 6b).

### TBARS content

**Fig. 7:**
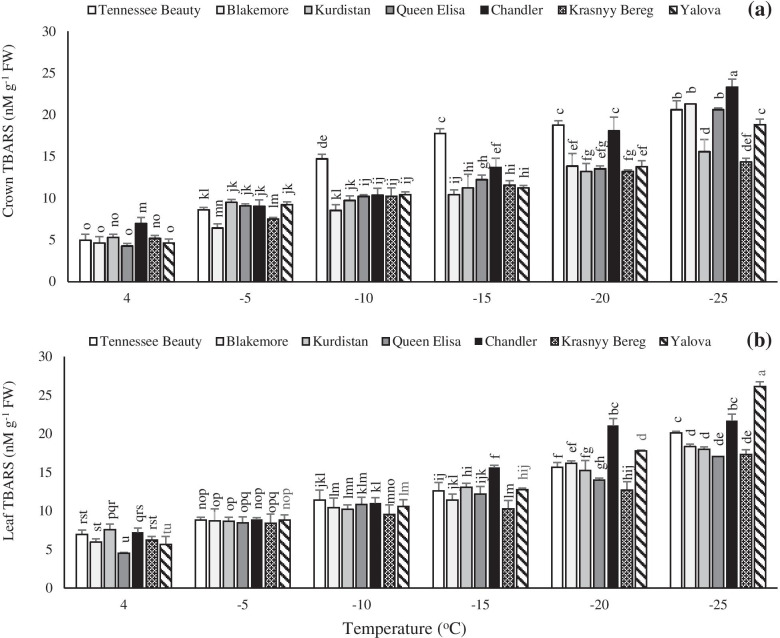
Effects of freezing temperature treatments on thiobarbituric acid reactive substances (TBARS) in the crown (**a**) and leaf (**b**) tissues of different strawberry cultivars. Data points are means of three replicates and vertical bars indicate standard deviations at *P ≤ 0.05*

The freezing temperature, to − 25 °C, caused a significant increase in the content of TBARS. In this regard, the lowest TBARS value was noted in the crown of cultivars Krasnyy Bereg and Kurdistan at − 25 °C (Fig. 7a). The highest and lowest increments were observed in the cultivars Queen Elisa and Krasnyy Bereg by 4.82 and 2.78 fold, respectively. The trend of changes in the TBARS content of crown in strawberry cultivars under freezing temperature treatments was similar to that in the leaves except in cultivar Tennessee Beauty. At − 25 °C, the highest leaf TBARS contents were observed in the cultivar Yalova followed by cultivar Chandler. However, the lowest value was detected in the cultivar Queen Elisa at − 25 °C (Fig. 7b). At temperatures below − 15 °C, the leaf TBARS content in the cultivars Yalova and Chandler showed a significant increase compared to tolerant cultivars Krasnyy Bereg and Queen Elisa (Fig. 7b).

### Activities of antioxidant enzymes

**Fig. 8:**
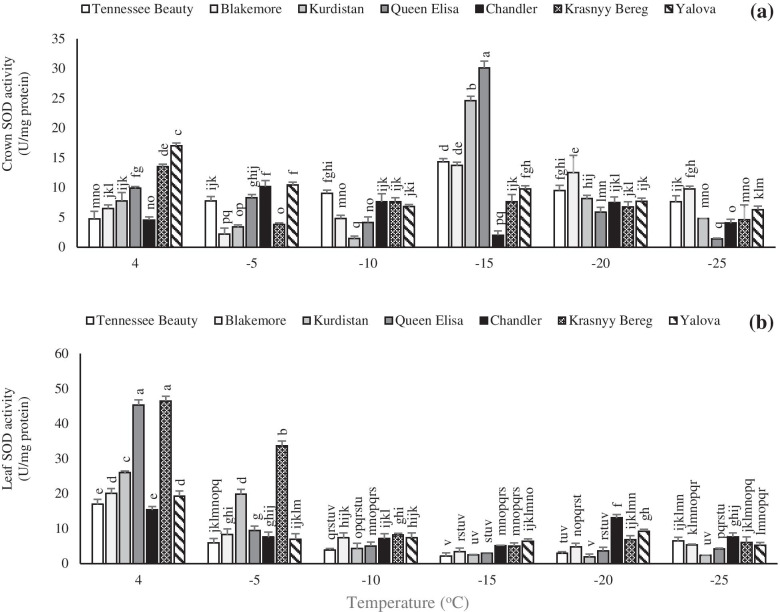
Effects of freezing temperature treatments on the activity of superoxide dismutase (SOD) in the crown (**a**) and leaf (**b**) tissues of different strawberry cultivars. Data points are means of three replicates and vertical bars indicate standard deviations at *P ≤ 0.05*

Freezing temperature treatments caused an overall increase in the SOD activity. However, variations in the crown SOD activity were different from that of leaf tissue. The increase of SOD activity in the crown occurred slightly late and the peak of its activity in most cultivars was noted at − 15 °C (Fig. 8a). The highest crown SOD activity was observed in tolerant cultivars Queen Elisa and Kurdistan. An increase in the leaf SOD activities was noted with a decrease in temperature in all the tested cultivars (Fig. 8b). The SOD activity was increased at + 4 °C and then gradually decreased with a decrease in the temperature. The highest leaf SOD activity was noted in the cultivars Krasnyy Bereg and Queen Elisa.

**Fig. 9:**
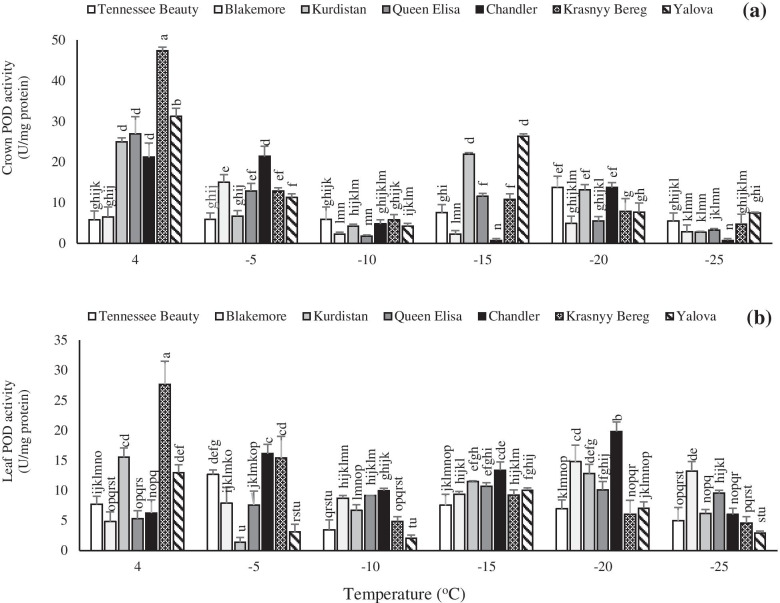
Effects of freezing temperature treatments on the activity of peroxidase (POD) in the crown (**a**) and leaf (**b**) tissues of different strawberry cultivars. Data points are means of three replicates and vertical bars indicate standard deviations at *P ≤ 0.05*

The crown tissue showed a faster response to POD activity in tolerant cultivars than the leaves. The secondary peak of POD activity was observed at − 15 °C in the cultivars Krasnyy Bereg, Queen Elisa, Kurdistan, and Yalova (Fig. 9a).

### Correlation and PCA analyses

**Fig. 10:**
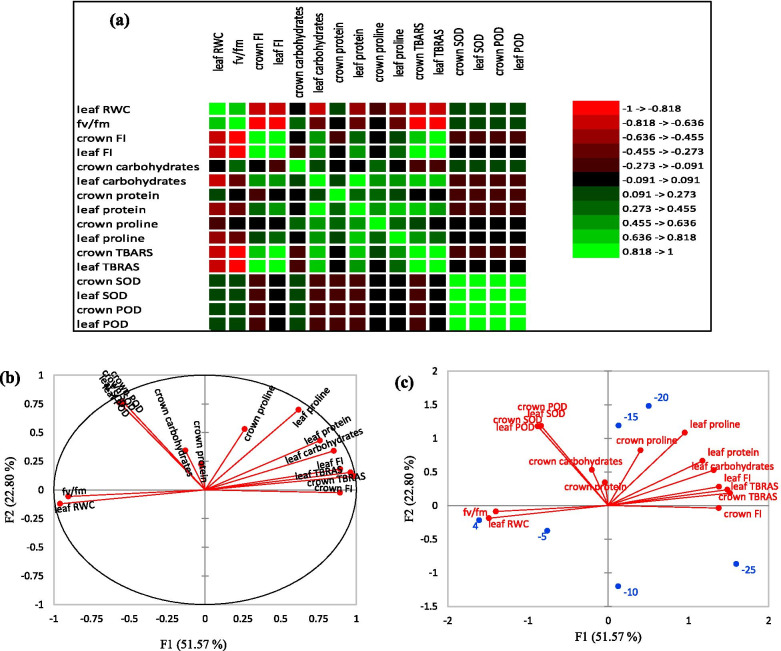
The correlation matrix between the different physiological and biochemical traits (**a**), principal component analysis (PCA) related to observations (**b**), and biplot (**c**) in strawberry cultivars under freezing temperature stress

Strong positive correlations were observed between the examined physiological and biochemical traits (Fig. 10a). There was a highly positive correlation between leaf FI and crown FI (*r* = 0.883). Leaf protein, crown proline, and leaf proline had a highly positive correlation with leaf carbohydrates. Leaf protein showed a high correlation with leaf proline (*r* = 0.716). On the other side, TBARS (in crown and leaf) had a strong positive correlation with crown FI (*r* = 0.816, 0.871) and leaf FI (*r* = 0.893, 0.909). Also, leaf TBARS showed a very high positive correlation with TBARS in the crown (*r* = 0.9).

Four principal components were accounted for 97.37% of the data variability (Table 2). The results of PCA indicated that PC1, the first principal component was accounted for 51.57% of total variation which exhibited significant positive correlations with leaf RWC, Fv/Fm, crown and leaf FI, crown and leaf TBARS. The first component was regarded as physiological and biochemical traits. PC2, the second principal component accounted for 74.37% of the total variation, SOD and POD in both leaf and crown tissues had higher Eigenvectors. The second component represents the contribution of enzymatic activity-related traits (Fig. 10b and c).

**Table 2:** Eigenvalue, variability, and cumulative variance percentage for the four factors resulted from principal component analysis (PCA)

	PC1	PC2	PC3	PC4
Eigenvalue	8.25	3.65	2.58	1.09
Variability (%)	51.57	22.8	16.15	6.85
Cumulative %	51.57	74.37	90.52	97.37

*PC1* the first principal component, *PC2* the second principal component, *PC3* the third principal component and *PC4* the fourth principal component

## Discussion

The results of the study supported the hypothesis as the tested strawberry cultivars significantly differed in their responses to low temperature stress. This variability and associated tolerance mechanism will help devise the programs to improve cold tolerance in different strawberry genotypes and improve its productivity in the temperature regions in particular.

The Freezing temperature tolerance of plant species is indirectly assessed by detecting their photosynthetic activity. Photosynthesis is a key plant metabolic process that is extremely sensitive to low temperature stress. There is evidence that the low temperature may disrupt the key components of photosynthesis [17]. The Fv/Fm ratio is an indicator of the maximum PSII photochemical efficiency and it can be used to examine the activity of PSII [18]. The low temperature induced membrane damage causes reduction in the PSII efficiency of sensitive cultivars. In this study, the observed reduction in Fv/Fm may be attributed to damage to the oxygen-evolving apparatus [19] and impairment of electron transfer within the PSII reaction resulting in PSII downstream under freezing temperature. The Fv/Fm remains in the range of 0.79 to 0.84 for many plant species, with a lowered value indicating plant stress condition [20]. In the healthy leaves, the value of Fv/Fm value remains closed to 0.8, independently of the studied plant species [20]. The lower value indicates that a proportion of PSII reaction centers are damaged due to a phenomenon called photo-inhibition often observed in plants under stress conditions. In this study, significant damage on the leaf at − 25 °C (Fv/Fm range from 0.4 to 0.71) appears to cause loss or stop of the PSII efficiency resulting in a significant decrease of Fv/Fm. There were obvious differences in the freezing induced decrease in Fv/Fm value between strawberry cultivars, ranging from a decrease by 50% in the cultivar Tennessee Beauty (freezing sensitive cultivar) to a decrease of 15% in the cultivar Krasnyy Bereg (freezing tolerant cultivar). The Fv/Fm values detected from plants under different regimes of light and temperature reached from 0.79 to 0.84 [21]. Choi et al. [21] reported that Fv/Fm values is lower in strawberry leaves under both, low temperature (5 °C) and low light conditions. In another study, Makaraci and Flore [22] stated that Chandler and Honeoye cultivars had lower values of Fv/Fm (0.772 and 0.806) than control (0.833) under − 2 °C temperature. Our observational evaluations showed that some leaves remained partially healthy in the center of the plant at − 25 °C, although the number of healthy leaves was higher in freezing-tolerant cultivars. In this study, the variable fluorescence reduction was not significant in freezing-tolerant cultivars, while a significant decrease was observed in freezing-sensitive cultivars.

The relative reduction in the tissue water contents is a part of the cold acclimation process and is crucial to freeze tolerance in strawberry plants [23]. Tissue dehydration during the cold acclimatization phase has been observed in many herbaceous plants as well as in various tree species [24]. Under the freezing stress, ice formation is generally initiated in the apoplastic spaces of the extra-cellular fluid which results in the movement of unfrozen water down the chemical potential gradient from inside the cell to the apoplastic space causing cellular dehydration and osmotic contraction of the cell [25]. In this study, leaf RWC was observed to follow a decreasing trend with a decrease in temperature which may be due to low temperature-induced osmotic stress. Upon exposure to cold stress, strawberry plants, like other plant species, experience low temperatures and as well low-temperature-induced drought stress [23]. Freezing first causes the formation of ice crystals in the plant vessels and then these crystals quickly reach all parts of the plant and penetrate the intercellular areas. These processes cause a difference in osmotic pressure outside and within the cells and lead to water leakage and severe plasmolysis of cells. This study indicates that the more severe decrease of leaf water, under freezing temperature, in tolerant cultivars may be closely linked with the openings of the stomata and preserving the possibility of entry of CO_2_ and is associated with the elimination of the stomata resistance in photosynthesis. The results of this experiment also demonstrated that tolerant cultivars are more capable to decrease rapidly the RWC. The low relative RWC in tolerant cultivars under low temperatures can also be due to the breakdown of large molecules such as polysaccharides and the production of simpler sugars which increases the osmotic potential and decreases the relative water contents [26, 27].

The lower FI in the tolerant cultivars may be due to the greater stability of the physical and chemical properties of the cell plasma membrane in both crown and leaf tissues of strawberry cultivars. The results of this study indicate that crown tissue displayed the lower FI than the leaf FI. The crown is especially sensitive to ice crystal-induced damages due to the large cells of the pith tissue. As described earlier by Campos et al. [28], cell membranes are likely to be the first areas to be affected by freezing damage. Low temperature stress reduces the fluidity of the membrane which in addition to lipid peroxidation, destroys the membrane and thus increases ion leakage. The degree of leaf damage is correlated with the damage to the plasma membrane of mesophilic cells [29]. The greatest damage is caused by intracellular freezing stress. However, Pearce [30], reported that the formed extracellular ice in both freezing tolerant and sensitive species may lead to cellular dehydration. However, freezing damage happens when the high dehydration is too much for the cells to tolerate. In this context, Azzarello et al. [31] indicated that freezing stress enhanced the membrane degradation of olive seedlings consequently that increased the ion leakage. Low temperature provokes an imbalance in photolysis resulting in an over-reduction of electron transport chain in thylakoid membranes and generation of reactive oxygen species (ROS) in PSI (photosystem I) and PSII [32, 33]. The production of ROS during the low temperature exposure also leads to lipid peroxidation resulting in ion leakage. Lipid peroxidation is a free radical-mediated degradation process that involves in homolysis of polyunsaturated fatty acids subsequent to the formation of lipid radicals.

In all tested cultivars, the trend of changes in the crown was different from the ones in the leaf in terms of soluble carbohydrates contents (Fig. 4). Significant variations in the soluble carbohydrates in the crown of strawberry cultivars indicate the sensitivity level of this tissue under freezing stress as well as the increased metabolic activity related to the transfer and consumption of carbohydrates and associated mechanisms involved in freezing temperature tolerance. The response of the tested cultivars to freezing temperatures was completely different in terms of leaf soluble carbohydrates changes. The cultivars Krasnyy Bereg, Queen Elisa, and Kurdistan cultivars showed more tolerance to low temperatures by having higher soluble carbohydrates content compared to other cultivars at − 25 °C. However, the upper levels of soluble carbohydrates in the tolerant cultivars may correspond to that of sensitive cultivars in some other plant species [11, 34–36]. The accumulation of various metabolites such as carbohydrates, in strawberry plants, is one of the known features of cold adaptation [11]. Soluble solids collaborate in increasing the osmotic potential and continuously decreasing the cytoplasmic freezing point [37]. Soluble sugars are involved in the crystallization process which protects plants from freezing damage [38]. Carbohydrates are elaborated in the formation of ice crystals in the intercellular space and reduction of the mechanical damage associated with freezing. When the temperature drops, the water freezes in the intercellular space, and the intracellular water is drawn from the cells to the intercellular ice mass. Cells of freezing tolerant organs accumulate low molecular weight carbohydrates and proline to prevent the dehydration of intracellular water [39]. Low temperature stimulates the expression of genes that encode the enzymes required for the biosynthesis of these compounds [37]. The cultivar Krasnyy Bereg had less changes in total soluble protein than other cultivars at temperatures − 20 °C. This indicates that there was no significant damage in this cultivar in the terms of protein degradation and reduction. While a sharp decrease was observed in total soluble protein in Tennessee Beauty cultivar at the temperature range of − 20 to − 25 °C. In all strawberry cultivars, the fluctuations of the total soluble proteins in the crown were more than the leaves, indicating more sensitiveness of the crown against freezing temperature. In general, the amount of crown soluble proteins was the same as the leaf amounts in sensitive cultivars, although the trend of changes in the crown was more severe and different. Crown tissue as the center of strawberry growth plays a key role in the metabolic activities related to the transportation and consumption of soluble proteins involved in freezing temperature tolerance. Increasing the content of soluble protein and carbohydrates is one of the properties of acclimation to cold stress and plays a major role in reducing freezing-induced damage in plant tissues. So that with decreasing temperature, the amount of protein often increases [36, 40]. Proteomic findings confirm that freezing tolerant proteins such as dehydrins and lipocalins accumulate in plasma membranes when plant exposure to low temperatures, although their action is unclear exactly. In this regard, Ouellet and Charron [37] demonstrated that these proteins protect molecules and cell structures from freezing damage and reduce the development of oxidative stress. Lukoševičiūtė et al. [11] also stated that the content of 18 kDa protein significantly increased during the cold adaptation process in the shoots in two strawberry cultivars.

The decrease in temperature also caused an overall increase in the amount of proline in both crown and leaf in all cultivars. The trend of changes of proline content in crown tissue was similar to changes in leaf. While the sensitiveness level of the crowns to freezing temperatures was higher than leaves. In addition, the peak of proline content happened faster in the crown tissue. The enhanced levels of proline accumulation in plants exposed to numerous abiotic stresses have been reported in various plant species [41, 42]. The present findings are consistent with other studies which found that proline has also been proposed as a non-water electron donor to PSII during abiotic stress conditions in plants besides playing the ROS-scavenger role [41, 43]. The increased proline accumulation in freezing sensitive strawberry cultivars indicates that these plants demonstrate the requirement of proline in the integrity of the cellular system for survival under stress conditions. The higher proline content and total soluble carbohydrates can be correlated with the ability of the strawberry plants to survive the freeze damage. According to Linden et al. [44], under low temperature conditions, the accumulation of soluble carbohydrates in winter wheat of the tolerant cultivar “Lina” was higher than the sensitive cultivar “Apollo”, while the amount of proline accumulated was higher in sensitive cultivars. Based on our results, the accumulation of soluble carbohydrates in the tolerant cultivars including Krasnyy Bereg, Queen Elisa, and Kurdistan was higher than sensitive cultivars such as Tennessee Beauty and Chandler under freezing temperatures, while its peak occurred faster in sensitive cultivars. Temperature below − 20 °C caused a significant decrement in proline accumulation of sensitive cultivars.

Determination of TBARS is often used as an indicator of the degree of peroxidation of membrane lipids and the level of plant sensitiveness to oxidative damage [45]. Overproduction of ROS has a detrimental effect on cell growth and development causing the peroxidation of membrane lipids and the production of toxic species such as TBARS, which leads to cell dysfunction and death [46, 47]. The reduction of unsaturated fatty acids leads to loss of fluidity and selective permeability of the membrane resulting in the reduced cold resistance [48]. According to the current results, the content of TBARS in leaves was generally higher in the tolerant cultivars. Reports showed that low temperature stress at different times led to an increment in malondialdehyde content in strawberry cultivars [49]. The present results are consistent with Palonen et al. [50] and Ershadi et al. [34] who showed that decreasing temperature often results in increasing the content of soluble carbohydrates and malondialdehyde.

In freezing-sensitive cultivars such as Tennessee Beauty and Chandler, the secondary peak of POD activity was observed by a delay at − 20 °C that represents a faster response of tolerant cultivars to accumulation of ROS for preventing oxidative stress injury under freezing temperatures. The leaf POD activity showed a considerable enhancement, especially in the tolerant cultivar Krasnyy Bereg than the other cultivars. Dissimilar to the SOD activity, POD activity showed a second peak at − 20 °C after the early peak at + 4 °C in all cultivars (Fig. 9b). Guo et al. [51] have been stated that there is a correlation between antioxidant enzyme activity and plant tolerance to abiotic stresses such as low temperature stress, so that plants with higher levels of antioxidants are more tolerant to oxidative damage. Luo et al. [52] observed that the activity of SOD rapidly increased at the beginning of cold treatment and then gradually decreased in two strawberry cultivars. Also, they observed that the POD showed a rapid enhancement at the onset of freezing treatment and then a steady upward trend during the freezing period, although a slight decrement was observed in one of them in the middle of the period. The higher activity of SOD in barley and rice cold tolerant cultivars than cold sensitive cultivars have been reported [53].

## Conclusions

In the present study, freezing stress was characterized by decreased Fv/Fm and RWC, and increased FI, TBARS, total carbohydrates, total proteins, proline content, and antioxidant enzyme activity. The extent of changes in abovementioned traits was cultivar dependent. According to the results, FI and TBARS were the best traits among destructive parameters for evaluating freezing tolerance. Moreover, maximum quantum yield of PSII (Fv/Fm index), as non-destructive parameters, showed a significant efficiency in rapid assessment for screening of freezing tolerant strawberry cultivars. Strawberry cultivar Krasnyy Bereg was the most tolerant to freezing temperatures compared to other cultivars, the cultivars Queen Elisa and Kurdistan were ranked next to it. These cultivars may be recommended for cultivation in regions with very low winter temperatures. In contrast to the other sensitive cultivars, the cultivar Yalova had good tolerance to freezing temperatures up to − 20 °C, but with decreasing the temperature to − 25 °C, the freezing damage increased significantly in this cultivar. The freezing-tolerant cultivars (Kurdistan, Queen Elisa, and Krasnyy Bereg) showed less level of freezing injury suggesting a higher tissues survival percentage.

Chlorophyll fluorescence measurement, as an easy and non-destructive field technique, had a significant efficiency in rapid assessment of invisible damages and maximum quantum yield of PSII (Fv/Fm index) was the best indicator compared to other methods for screening of freezing-tolerant genotypes. The cultivars with a high level of tolerance to freezing temperature can be used as a parent in breeding programs to produce the new cultivars.

## Methods

### Growth conditions and temperature treatments

Bare-root daughters of seven strawberry cultivars (*Fragaria × ananassa* Dutch.) with uniform crown diameter (10 mm) were provided by the Kurdistan Agricultural and Natural Resources Research and Education Center, AREEO, Kurdistan, Iran. Experimental research and field studies on strawberry cultivars complied with Iran and Kurdistan province local legislation. The breeding parents and geographical origin of the tested cultivars (Tennessee Beauty, Blakemore, Kurdistan, Queen Elisa, Chandler, Krasnyy Bereg, and Yalova) are given in Table 3. Strawberry plants were grown in pots filled with perlite, vermicompost, and coco peat (60:30:10) and were maintained outdoor during March–October in 2019 until the imposition of freezing temperature treatments. Monthly weather conditions of the experimental site are given in Table 4. The plants were exposed to the natural low temperatures of autumn for cold acclimation. For the imposition of freezing temperature treatments, the potted plants were exposed to the temperature of + 4 (as control), − 5, − 10, − 15, − 20, and − 25 °C by a programmable thermogradient refrigeration chamber (Thermogradient cooling chamber made by Kimia Rahavard Company, Iran) equipped with halogen lightbulb and the light intensity of ≈300 μmol m^− 2^ s^− 1^ was maintained during the light period of 12 h and 70 ± 5% relative humidity. Before the imposition of freezing temperature treatments, leaves of strawberries were thoroughly washed with distilled water to remove surface contaminants and were allowed to dry naturally to avoid water residue (later ice) formation on their surface. Temperature treatments were applied based on the average ambient temperature and considering the process of acclimatization of plants to freezing and adapting to natural conditions as much as possible as follows: + 4 °C (October 30), − 5 °C (November 20), − 10 °C (December 4), − 15 °C (December 20), − 20 °C (January 15), and − 25 °C (January 30). The process of gradual decrease of temperature continued at a rate of 2 °C/hour until the target temperature was reached (for example, − 20 °C). The samples were then exposed to the target temperatures for 75 min. Afterward, the temperature was gradually increased again (at a rate of 2 °C/hour), until the temperature + 4 °C was reached. Four strawberry plants of each cultivar (one plant in each pot) per three replicates were evaluated in each experimental unit. Three individual crowns and leaves were taken from plants for further analyses. For chlorophyll fluorescence assessment, the samples were selected 1 week after the imposition of temperature treatments to precisely assess the damage to the green tissue.

**Table 3:** The breeding parents and origin of strawberry cultivars used in the study

Cultivar	Breeding parents	Origin
Tennessee Beauty	Howard 17 × Missionary	USA
Blakemore	Missionary × Howard 17	USA
Kurdistan^a^	Unknown	Unknown
Queen Elisa	Miss. × Usb 35	Italy
Chandler	Douglas × Cal 72–361-105	USA
Krasnyy Bereg	Venta × Tenira	Belarus
Yalova	Arnavutkoy ×Aliso	Turkey

^a^A genotype cultivated in Kurdistan Province, Iran since more than 50 years

**Table 4:** The weather data of the experimental site during the course of the investigation

Month	Average min temp. (°C)	Average max temp. (°C)	Mean temp. (°C)	Average relative humidity (%)	Average min humidity (%)	Average max humidity (%)	Total precipitation (mm)
Apr	3.60	18.7	11.2	55.0	28.9	81.1	39.6
May	7.90	27.4	17.7	38.2	13.6	62.7	7.80
Jun	13.1	34.4	23.8	27.4	9.70	45.2	0.00
Jul	18.7	39.2	28.9	22.0	8.40	35.6	2.00
Aug	18.2	39.6	28.9	19.3	6.80	31.8	0.00
Sep	14.3	33.8	24.1	33.4	12.6	54.2	5.50
Oct	9.60	28.7	19.1	36.5	15.7	57.4	1.10
Nov	4.50	15.7	10.1	69.5	48.1	90.9	206.4
Dec	−1.4	8.40	3.50	70.1	51.3	88.9	40.6
Jan	−2.2	8.50	3.20	65.9	46.1	85.7	29.6
Feb	−2.9	9.80	3.50	62.6	38.8	86.5	56.9
Mar	3.00	16.6	9.80	55.3	30.8	79.9	40.8

### Biochemical and physiological parameters

#### Maximum quantum yield of PSII (Fv/Fm)

Three middle mature leaves, from each pot, were selected and chlorophyll fluorescence was measured by a portable chlorophyll fluorometer (OS-30P, Opti-Sciences Inc., USA). The measurement was done after the plants were dark-adapted for 20 min. The maximum quantum efficiency of Photosystem II (PSII) primary photochemistry (Fv/Fm) was calculated following Miralles-Crespo et al. [54].

#### Leaf relative water content (RWC)

Leaf relative water content (RWC) were determined from five randomly selected fully developed leaves. The RWC was calculated using the following equation (Eq. 1):

1 $$\mathrm{RWC}=\left(\mathrm{FW}-\mathrm{DW}\right)/\left(\mathrm{TW}-\mathrm{DW}\right)\times 100$$

Where FW, DW, and TW refer to fresh weight, dry weight, and turgid weight of leaf, respectively.

#### Freezing injury (FI)

Three samples of the crown and leaf tissues were put into tubes containing distilled water. The tubes were placed on a shaker at 120×*g* for 24 h at room temperature. The initial electrical conductivity (EC) was measured by an electrical conductivity meter for tissues placed at + 4 °C for 5 h. Final electrical conductivity was measured after autoclaving at 120 °C for 30 min, followed by incubation at room temperature for 20 h. Percent of injury was calculated based on [55] as following equation (Eq. 2):

2 $$\mathrm{Injury}\ \left(\%\right)=\left(\%{\mathrm{EL}}_{\Big(\mathrm{T}}{\circ}_{\mathrm{C}\Big)}-\%{\mathrm{EL}}_{\Big(4}{\circ}_{\mathrm{C}\Big)}\right)/\left(100-\%{\mathrm{EL}}_{\Big(4}{\circ}_{\mathrm{C}\Big)}\right)\times 100$$

Where % EL (_T °C_) and % EL (_4 °C_) are the percent of electrolyte leakage (EL) values based on initial electrical conductivity over total electrical conductivity for each freeze target temperature (T °C) and unfrozen control (4 °C), respectively. The injury data were transformed using a method proposed by Lim et al. [56].

### Total soluble carbohydrates and total soluble protein

The total soluble carbohydrates in the crown and leaf tissues were estimated by a spectrophotometer (SPECORD model 210, Analytik Jena, Germany) as described by Yemm and Willis [57]. The total soluble protein was measured in the crown and leaf tissues according to Bradford method [58].

### Proline and TBARS contents

The proline content in the crown and leaf tissues was determined according to Bates et al. [59]. The TBARS **(**ThioBarbituric Acid Reactive Substances) contents were examined based on the formation of thiobarbituric acid complex formed by malondialdehyde (MDA) complex [60].

### Antioxidant enzyme activities

The activity of superoxide dismutase (SOD) was determined by measuring its ability to prevent optical reduction of the nitro blue tetrazolium chloride following Beauchamp and Fridovich [61]. The peroxidase (POD) activity was estimated based on the decomposition of H_2_O_2_ into water and oxygen as described by Hemeda and Klein [62].

### Statistical analysis

The experimental data were analyzed by the analysis of variance (ANOVA) using statistical package SAS 9.4 (SAS Institute Inc., ver. 9.1, Cary, NC, USA). For mean separation, Duncan’s new multiple range test was used. Computation of Pearson correlation coefficients and principal component analysis (PCA) was done using XLSTAT to investigate the degree of association between the features examined under low temperature stress.

## Supplementary Information

**Additional file 1: Figure S1.** Freezing injury in crown and leaf of Yalova cultivar under freezing temperature treatments (− 15, − 20, and − 25 °C).

## Data Availability

The data that support the findings of this study are available from the corresponding author upon reasonable request.

## Abbreviations

RWC: Relative Water Content
TBARS: ThioBarbituric Acid Reactive Substances
FI: Freezing Injury
SOD: Superoxide Dismutase
POD: Peroxidase
