# Supplementary material for: Physiological and biochemical responses of strawberry crown and leaf tissues to freezing stress
Source: BMC Plant Biol. 2021 Nov 13;21:532. doi: 10.1186/s12870-021-03300-2 (PMC8590311; doi:10.1186/s12870-021-03300-2)
Supplement: Supplementary file 1 — Additional file 1: Figure S1. Freezing injury in crown and leaf of Yalova cultivar under freezing temperature treatments (− 15, − 20, and − 25 °C). [file 12870_2021_3300_MOESM1_ESM.docx]

**Physiological and biochemical responses of strawberry crown and leaf tissues to low temperature stress**

**Elnaz Zareei^1^, Farhad Karami^2^*, Mansour Gholami^3^, Saber Avestan^2^, Rishi Aryal^4^, Gholamreza Gohari^5^, Muhammad Farooq^6^**

^1^ Department of Horticultural Science, Faculty of Agriculture, University of Kurdistan, Sanandaj, Iran

^2^ Horticultural Research Department, Kurdistan Agricultural and Natural Resources Research and Education Center, AREEO, Sanandaj, Iran

^3^ Department of Horticultural Sciences, Faculty of Agriculture, Bu-Ali Sina University, Hamedan, Iran

^4^ Department of Horticultural Science, NC State University, Raleigh, North Carolina, USA

^5^ Department of Horticultural Sciences, Faculty of Agriculture, University of Maragheh, Maragheh, Iran

^6^ Department of Plant Sciences, College of Agricultural and Marine Sciences, Sultan Qaboos University, Al-Khoud 123, Oman

^*^ Corresponding author’s email address: [f.karami@areeo.ac.ir](mailto:f.karami@areeo.ac.ir)

*
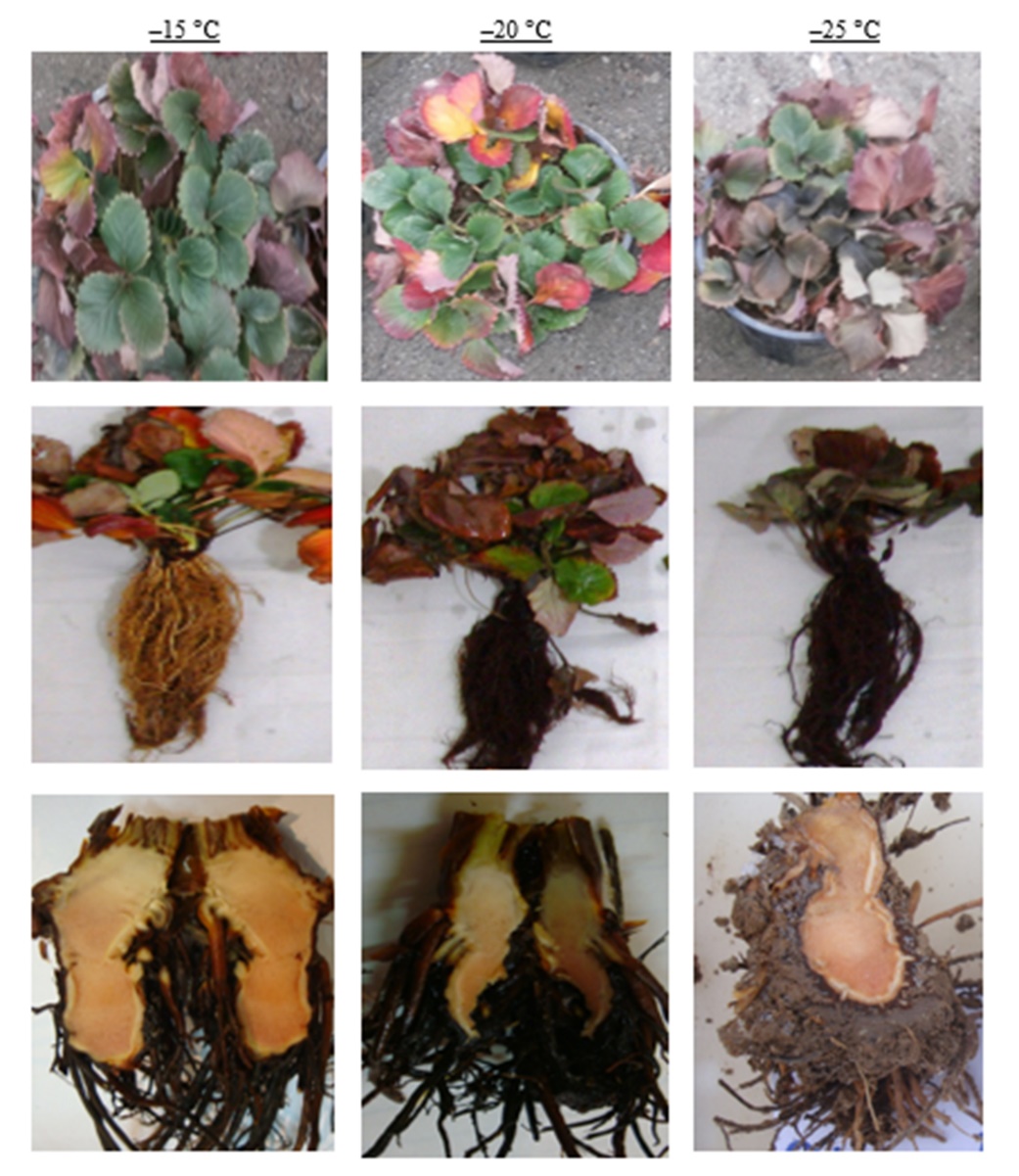
*

**Figure S1.** Freezing injury in crown and leaf of Yalova cultivar under low temperature treatments (–15, –20, and –25 °C)
